# Job vacancy at the European Centre for Disease Prevention and Control 

**DOI:** 10.2807/1560-7917.ES.2017.22.9.30471

**Published:** 2017-03-02

**Authors:** Eurosurveillance editorial team

**Affiliations:** 1 European Centre for Disease Prevention and Control (ECDC), Stockholm, Sweden

The European Centre for Disease Prevention and Control (ECDC) invites applications for the following position:

**Expert Hepatitis B/C **

The deadline for application is 27 March 2017. For more information, visit the ECDC website: http://ecdc.europa.eu/en/aboutus/jobs/Pages/JobOpportunities.aspx

